# Comparison of Different Sets of Features for Human Activity Recognition by Wearable Sensors

**DOI:** 10.3390/s18124189

**Published:** 2018-11-29

**Authors:** Samanta Rosati, Gabriella Balestra, Marco Knaflitz

**Affiliations:** Department of Electronics and Telecommunications, Politecnico di Torino, 10129 Torino, Italy; gabriella.balestra@polito.it (G.B.); marco.knaflitz@polito.it (M.K.)

**Keywords:** human activity recognition, wearable sensors, MIMU, genetic algorithm, feature selection, classifier optimization, machine learning

## Abstract

Human Activity Recognition (HAR) refers to an emerging area of interest for medical, military, and security applications. However, the identification of the features to be used for activity classification and recognition is still an open point. The aim of this study was to compare two different feature sets for HAR. Particularly, we compared a set including time, frequency, and time-frequency domain features widely used in literature (*FeatSet_A*) with a set of time-domain features derived by considering the physical meaning of the acquired signals (*FeatSet_B*). The comparison of the two sets were based on the performances obtained using four machine learning classifiers. Sixty-one healthy subjects were asked to perform seven different daily activities wearing a MIMU-based device. Each signal was segmented using a 5-s window and for each window, 222 and 221 variables were extracted for the *FeatSet_A* and *FeatSet_B* respectively. Each set was reduced using a Genetic Algorithm (GA) simultaneously performing feature selection and classifier optimization. Our results showed that Support Vector Machine achieved the highest performances using both sets (97.1% and 96.7% for *FeatSet_A* and *FeatSet_B* respectively). However, *FeatSet_B* allows to better understand alterations of the biomechanical behavior in more complex situations, such as when applied to pathological subjects.

## 1. Introduction

Human Activity Recognition (HAR) is a growing research field of great interest for medical, military, and security applications. Focusing on the healthcare domain, HAR was successfully applied for monitoring and observation of the elderly [1], remote detection and classification of falls [2], medical diagnosis [3], rehabilitation and physical therapy [4].

A HAR system is usually made up of two components: (1) a wearable device, equipped with a set of sensors (i.e., accelerometers, gyroscopes, magnetometers,…) suitable for capturing human movements during daily life, and (2) a processing tool that recognizes the activity performed in a given instant by the subject. The most common systems employed for HAR are miniature magnetic and inertial measurement units (MIMUs) that work only as a data logger, performing the signal acquisition and storage, while an external system (pc, tablet, smartphone) is needed to process signals and recognize the activities. However, for all those applications in which a real-time feedback is required, it is important to create a stand-alone device able both to acquire several magnetic-inertial signals for long periods of time and to identify the performed activities as fast as possible. From this perspective, the desired device should be lightweight, small and easy to be worn from the subject, provided with a long-lasting battery, and equipped with a microcontroller having enough internal memory for signals and activities storage and able to support the implementation of a classifier for activity recognition. From the classifier point of view, it should be as fast as possible, in order to return a real-time feedback, with low storage requirements and easy to be realized on a microcontroller. In a previous work, we showed that a suitable machine learning classifier is Decision Tree (DT), that is also able to reduce the number of input variables, decreasing the computational time, even if its accuracy was lower than other methods [5].

Another challenging and still open aspect when dealing with HAR is the identification of the correct set of input variables (or features) for the classifier. Analyzing the literature, different approaches can be found. The most popular approach is based on time-domain features [6,7,8], that are usually of a statistical nature: mean value, median, variance, skewness, kurtosis, percentiles and interquartile range. Some studies use cross-correlation coefficients to quantify the similarity between signals coming from different axes [9,10], but other studies demonstrated the inefficiency of these features [11]. To give an idea of the energy and power contained in signals, frequency-domain features, such as signal power, root mean square value, auto-correlation coefficients, mean and median frequency, and spectral entropy, are commonly extracted [8,12]. Finally, some approaches based on the time-frequency domain can be found in the literature, in particular using the Discrete Wavelet Transform (DWT), that allows a decomposition of signals into several coefficients, each containing frequency data across temporal changes [13]. A detailed review of features used for HAR applications and belonging to time, frequency and time-frequency domains can be found in [14]. Although the great majority of applications use these kinds of features, three main problems must be addressed: (1) the extraction of frequency-domain and wavelet-domain features could result really hard for a microprocessor [15]; (2) the great majority of these features are not directly related to the acquired signals and, thus, they are difficult to attribute to physical quantities, complicating the interpretation of the results and the understanding of errors; (3) the number of variables proposed in the literature is huge and this is not always associated with high classification accuracy since some of them could be sources of noise [16]. 

Feature Selection (FS) is a fundamental step when dealing with high-dimensional data, allowing for eliminating those variables that are redundant or irrelevant for the system description. Moreover, it has been proven that FS increases the classification performance [17], due to the removal of those variables introducing noise during the classifier construction and application. Two main categories of FS algorithms have been proposed in the literature and successfully applied in the biomedical field for dataset [18], signal [19] and image [20] processing: filter and wrapper methods [21]. Filter methods perform FS independently of the learning algorithm: variables are examined individually to identify those more relevant for describing the inner structure of the analyzed dataset. Since each variable is considered independently during the selection procedure, groups of features having strong discriminatory power may be ignored. Conversely, in wrapper methods, the selection of the feature subset is performed simultaneously with the estimation of its goodness in the learning task. For this reason, this latter category of FS methods usually can reach better performances than filter methods [21], since it allows for exploring also feature dependencies. On the other hand, wrapper FS could be computationally very intensive and the obtained feature subset optimized only for the specific learning algorithm or classifier. 

Moreover, once a feature subset is fixed, different classification results might be obtained changing the classifier parameters, since they strongly influence the classification performance [22]. Several approaches have been developed for parameters tuning, e.g., grid search, random search, heuristic search [23]. However, the simultaneous selection of the optimal feature subset and optimization of the classifier parameters is likely the only way assuring to reach the best performances. Since an exhaustive search of the best couple feature subset-classifier parameters is unfeasible in most real situations, heuristic search represents a convenient way to find a good compromise between reasonable computational time and sub-optimal solutions. In particular, genetic algorithms (GAs) have been applied for solving optimization problems connected to FS [17] and parameter tuning [22], but very scarce applications can be found for the simultaneous optimization of both aspects.

The aim of this study is to compare two sets of features for real-time HAR applications: *FeatSet_A* comprising time, frequency and time-frequency domain parameters presented in the literature and *FeatSet_B* consisting of variables belonging only to the time-domain and derived from the understanding of how a specific activity will affect the sensor signals. The most informative features for each set were identified using a GA that simultaneously performs feature selection and optimization of the classifier parameters. Then, the obtained feature subsets were compared analyzing the performances reached by four different machine learning classifiers.

The rest of the paper is divided as follows: Section 2 describes related works about HAR using wearable sensors. Section 3 presents the protocol and population involved in our experiment, the extracted features and the GA used for simultaneous FS and classifier optimization. Results are presented in Section 4 and discussed in Section 5. Section 6 concludes this study and proposes future directions in this context.

## 2. Related Work

A huge number of studies was proposed in the literature for HAR by means of wearable sensors. Although an exhaustive analysis of publications dealing with these aspects is beyond the scope of this paper (a recent review can be found in [24]), several aspects can be used to characterize and summarize these studies, such as acquired signals, extracted features and algorithms used for dimensionality reduction and activity recognition. 

Accelerometric signals are common to all HAR applications. Some studies used this information alone [25,26,27], but more often accelerometers were combined with gyroscopes [12,28,29] and magnetometers [30,31]. In few cases other signals were taken into account such as quaternions [32], temperature [1], gravity [33] or data acquired from ambient sensors [1].

Once they were acquired, the raw signals were rarely employed as they are [33,34] but usually some kind of processing was applied to extract a set of informative features. In general, most of extracted features belongs to the time-domain (e.g., mean, standard deviation, minimum value, maximum value, range,…) and the frequency-domain (such as mean and median frequency, spectral entropy, signal power, entropy) [32,35,36]. However, other different variables can be found in literature, such as time-frequency domain variables used in the studies by Eyobu et al. [12] and Tian et al. [37], or the cepstral features proposed by San-Segundo et al. [26] and Vanrell et al. [38].

Regarding the dimensions of the obtained feature sets, three different approaches were followed in the literature. In some studies no dimensionality reduction was performed and, thus, the whole set of variables was used for the recognition phase [39,40]. The second approach achieves dimensionality reduction by means of a transformation of the original set of variables in a new one with lower dimensionality. The most common method belonging to this category is the principal component analysis (PCA), that was employed for example in ref. [41,42]. Finally, different FS methods were used to reduce the number of variables without any transformation, such as Minimum Redundancy Maximum Relevance [43], recursive feature elimination [34], Information Gain [25], or evolutionary algorithms [44].

Since the aim of a HAR application is to identify the performed activity, a proper learning algorithm must be applied as final step. The great majority of the studies in this fields was based on supervised learning algorithms, ranging from machine learning (support vector machine [33], decision tree [27], random forest [32], multilayer perceptron [44],…) to the emerging deep learning neural networks [45,46,47]. However, sporadic applications of unsupervised learning algorithms were proposed [48]. Ensemble learning, that combines different classifiers to improve the final performances, was proposed by Tian et al. [37] and Garcia-Ceja et al. [40].

## 3. Materials and Methods

### 3.1. Signal Acquisition and Experimental Setup

**Figure 1:**
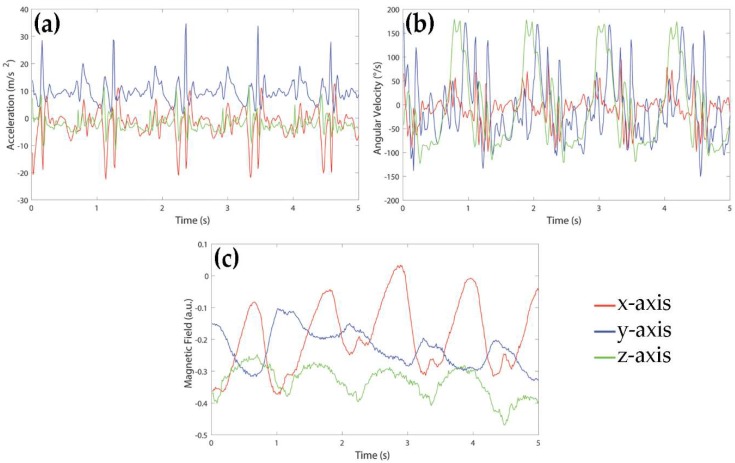
Example of signals acquired by (**a**) accelerometer, (**b**) gyroscope and (**c**) magnetometer during 5 s of walking of a healthy subject.

Signals were acquired using a MIMU-based device by Medical Technology (Torino, Italy). The sensor unit consisted of a tri-axial accelerometer, a tri-axial gyroscope and a tri-axial magnetometer allowing for acquiring acceleration, rate of turn, and Earth-magnetic field data, for a total of nine signals. The measurement range was ± 4 g for the accelerometers, ± 2000°/s for the gyroscopes and ± 4 G for the magnetometers. The sampling frequency of all signals was 80 Hz. An example of signals acquired during a walk of a healthy subject is shown in Figure 1. For the purpose of this study, signals were recorded in local data storage devices and transmitted to a laptop for the following analysis.

The MIMU sensor was located on the lateral side of the right thigh. The *y*-axis was oriented in down-top vertical direction, *x*-axis was aligned to the antero-posterior direction, and *z*-axis was aligned to the medio-lateral direction, pointing to lateral side. Sixty-one young and healthy subjects (28 males, 33 females; age: 22 ± 2 years; age range: 20–28 years; height: 169.9 ± 8.3 cm; weight: 64.3 ± 11.0 kg) with no history of physical disabilities or injuries were involved in this study. All subjects were asked to perform seven simple activities: resting (A_1_, comprising sitting and laying), upright standing (A_2_), level walking (A_3_), ascending and descending stairs (A_4_ and A_5_), uphill and downhill walking (A_6_ and A_7_). All activities lasted 60 s and were repeated five times by each subject. Activities were executed in indoor and outdoor areas, following a default path, without any speed restriction and style of performing. Each subject signed an informed consent form. Since this was an observational study and subjects were not exposed to any harm, the study protocol was not submitted to an ethical committee nor to an institutional review board.

### 3.2. Dataset Construction and Feature Extraction

To avoid bias due to the magnetic direction of the performed activities during signals acquisition and magnetic disturbances on the magnetometer, only inertial information (i.e., accelerometer and gyroscope signals) was used for HAR. Each signal was segmented using a 5 s sliding window with an overlap of 3 s between subsequent windows. The total number of processed windows was included in the validation set while the training set was obtained by randomly selecting 10% of windows for each activity of each subject. 

For every window, two sets of features were extracted: *FeatSet_A,* comprising features commonly used in literature, and *FeatSet_B*, containing only time-domain features derived from the analysis of the expected biomechanical effect of a given activity on the sensor signals. Since features included in the two sets had different ranges, the min-max scaling method was applied to the training sets to obtain variables between 0 and 1:

(1) $$Var\_norm_{i}=\frac{Var_{i}-\min\left(Var_{i}\right)}{\max\left(Var_{i}\right)-\min\left(Var_{i}\right)}$$

where $Var_{i}$ is the original value of the *i*-th variable.

Even if standardization using mean and variance of each variable is commonly used for machine learning purposes, it is suitable where close-to-Gaussian distribution could be assumed and might be inappropriate for very heterogeneous features [49]. In this study we used features belonging to very different domains, thus we preferred to use the min-max scaling that also preserves the original value distribution of each variable.

Finally, since all machine learning methods tested in this study were supervised methods, each window in the training and validation sets was labeled with the activity performed by the subject in that specific moment. In particular, an integer number was used to codify each activity, ranging from 1 to 7 for activities from A_1_ to A_7_, respectively.

#### 3.2.1. *FeatSet_A*

*FeatSet_A* included 222 features belonging to different domains. In particular, for the six considered signals we calculated:

- 20 time-domain features [14,50,51] (mean value, variance, standard deviation, skewness, kurtosis, minimum and maximum values, 25th and 75th percentiles, interquartile range, 10 samples of the autocorrelation sequence);
- three frequency-domain features [14,50,51] (mean and median frequency of the power spectrum, Shannon spectral entropy);
- 14 time-frequency domain features [14] (norms of approximation and detail coefficients, considering seven levels of decomposition of the discrete wavelet transform).

#### 3.2.2. *FeatSet_B*

A set of 221 features was extracted based on the time-domain analysis of the signals. First, we defined the positive and negative peaks as the maximum and the minimum values reached between two consecutive zero crossings, respectively. Then, we calculated the following 33 features for the six signals:

- number of zero crossing (one feature);
- number of positive and negative peaks (two features);
- mean value, standard deviation, maximum, minimum, and range of duration for positive, negative, and total peaks (15 features);
- mean value, standard deviation, maximum, minimum, and range of time-to-peak for positive, negative, and total peaks (15 features).

Moreover, we computed single and double integration of the acceleration in the antero-posterior and vertical directions, and the single integration of the rate of turn in medio-lateral direction. These signals represented the velocity and distance traveled by the limb in the corresponding directions. Other 23 features were extracted from these signals:

- mean of the single and double integration of vertical acceleration (two features);
- mean and RMS value of single integration of antero-posterior acceleration (two features);
- RMS value of double integration of antero-posterior acceleration (one feature);
- number of positive, negative and total peaks of the single integration of the rate of turn in medio-lateral direction (three features);
- mean value, standard deviation, maximum, minimum, and range of duration of positive, negative and total peaks of the single integration of the rate of turn in medio-lateral direction (15 features).

### 3.3. Recognition of Static Activities

**Figure 2:**
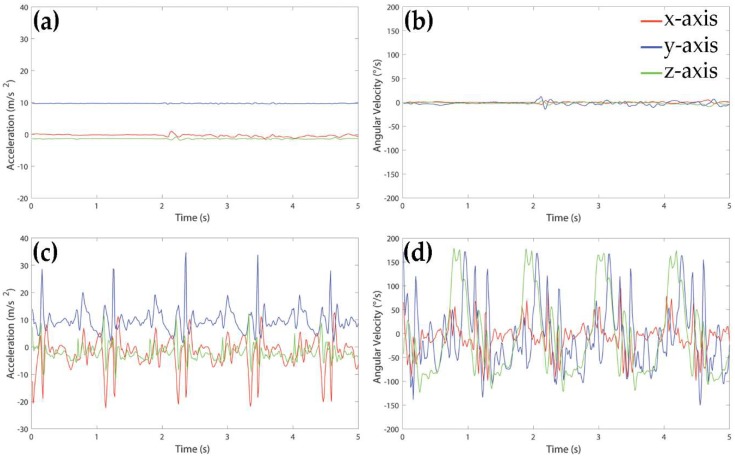
Example of signals acquired by accelerometer (**left panels**) and gyroscope (**right panels**) during 5 s of upright standing (panels (**a**,**b**)) and walking (panels (**c**,**d**)) of a healthy subject.

Since static (resting and upright standing) and dynamic activities (level walking, ascending and descending stairs, uphill and downhill walking) showed very different types of behavior from the signal point of view, we decided to implement a first recognition step, based on a couple of rules, to discriminate these two classes of movements. Figure 2 shows an example of accelerometer and gyroscope signals acquired during upright standing (panels a and b) and walking (panels c and d) of a healthy subject.

The following rule was used to separate windows representing static activities from those associated to dynamic activities:

*if* variance of gyroscope signal in *z* direction *is* below 600 deg∙s^−1^, *then* window represents a static activity, *else* window represents a dynamic activity.

Windows recognized as static activities were further separated between resting and standing windows according to the following rule:

*if* mean of accelerometer signal in *y* direction *is* below 8.5 m∙s^−2^, *then* the window is classified as resting, *else* the window is classified as standing.

All windows recognized as dynamic activities were pooled together and used for the following step of HAR based on GA and machine learning classifiers. 

### 3.4. Genetic Algorithm for Simultaneous Feature Selection and Classifier Optimization

GA [52] is a well-known optimization algorithm belonging to the class of metaheuristics, i.e., algorithms designed to search for optimal solutions of a given optimization problem in a reasonable time. A GA is inspired to the Darwin’s theory of evolution and, as such, it evolves a population of possible solutions (or individuals) toward better solutions, using the genetic operators of mutation and crossover. The main steps of a generic GA can be summarized as follows:

(1) Generation of an initial population: a random pool of individuals is generated by the algorithm, where an individual is represented by a binary vector, and the fitness value for all of them is calculated. The fitness function is a mathematical function that measures the goodness of a specific individual to solve the optimization problem.
(2) Parents’ selection: a subset of individuals is selected to be parents of a new generation of solutions by means of a selection operator. The most used operator is the roulette wheel.
(3) Application of genetic operators: a new generation of individuals is obtained by applying mutation and crossover to the parents. Mutation produces a change in one or more bits of a solution and it is used for maintaining genetic diversity from one generation to the next. The bits to be mutated are randomly selected according to a mutation probability (usually very low, from 0.1 to 0.2). By mutation, a “1” bit in the original solution becomes a “0” bit and vice-versa. Crossover is applied to a couple of individuals with the aim of combining their genetic information. The two individuals are cut in correspondence of one or more random cut-points and the produced substrings are exchanged between them. Each individual has a crossover probability (usually higher than 0.8) to be part of at least one couple.
(4) Termination: if the stopping condition is not reached, a new population of individuals is selected among children and parents and the algorithm restarts from Step 2. The stopping condition is usually based on a given number of iterations or to a plateau in the fitness values of the new generations.

In this study we developed an ad-hoc GA for searching the optimal feature subset and classifier parameters, simultaneously. Four classifiers belonging to machine learning were tested and optimized: K-Nearest Neighbors (KNN), Feedforward Neural Network (FNN), Support Vector Machine (SVM) and Decision Tree (DT). Since all couples classifier-feature set were optimized, a total of 8 GAs were implemented (four classifiers × two feature sets). 

Each solution was represented by a binary vector made up of two concatenated substrings: a first substring used for the selection of the most informative features to be input in the classifier and a second substring codifying the classifier parameters. For the first substring, we associated one bit to each available feature, obtaining a number of bits equal to the total number of features included in the considered feature set (*FeatSet_A* or *FeatSet_B*). A bit assuming value equal to “1” identified a feature included in the subset and used by the classifier, while a “0” labelled a not-used feature. The number of bits constituting the second substring was defined for each specific classifier, according to the number of parameters to be optimized and the values we wanted to explore. The details of the codification scheme used for the second substring can be found in the following sections for each classifier tested in this study.

The initial population of possible solutions comprised 400 individuals. The fitness function of each solution was measured according to the following equation:

(2) $$fitness=1-acc+0.3\times \left(\underset{\forall activity}{\max}\left(acc_{activity}\right)-\underset{\forall activity}{\min}\left(acc_{activity}\right)\right)$$

where the total accuracy (*acc*) and the accuracy for the *i*-th activity ($acc_{activity}$) were calculated on the validation set for each specific classifier and were expressed in percentage between 0 and 1. The classifiers were trained using the training set, fed with the feature subset defined by the first substring and set up with the parameters codified in the second substring. In Equation (2), the first part of the formula aims at maximizing the classifier performances and the second part is a penalty term introduced to balance the performances among the different classes. Lower fitness values are associated with better solutions.

The parents’ selection was based on the roulette wheel algorithm [52], in which the probability of each individual to be selected as parent is proportional to its fitness: individuals with better fitness values have higher probability to become parents of the new generation.

Crossover was implemented with four random cut-points and probability equal to 1. The mutation probability was set to 0.2. Two stopping conditions were implemented: maximum number of iterations (experimentally established as 30) and a plateau in the best fitness value for 15 consecutive iterations. All GAs and classifiers were implemented in Matlab2018a^®^ (The MathWorks, Natick, MA, USA) environment.

#### 3.4.1. K-Nearest Neighbors

KNN algorithm is a simple classification algorithm based on the calculation of the distance (usually the Euclidean distance) between the new element to be classified and the elements in the training set. Firstly, the training elements are sorted in descending order according to their distance from the new element. Then, the most frequent class of the first K elements (called neighbors) is associated to the new element.

For this kind of classifier, only the value of the K neighbors must be decided. A common starting value for K is $K_{in}=\sqrt{N}$ [53], where N was the number of elements in the training set. Beginning from this consideration, we decided to analyze 32 values around $K_{in}$ and, thus, we used five bits for the second substring of each GA solution (2^5^ = 32): each possible value assumed by the second substring was associated to a specific K value to be set in the classifier.

#### 3.4.2. Feedforward Neural Network

A FNN is made up of a set of neurons, connected by weighted arcs, that process the input information according to the McCulloch and Pitts model [54]:

(3) $$y=f\left(\underset{i}{\sum }w_{i}\cdot x_{i}\right)$$

where y is the output of the neuron, $w_{i}$ are weights of the incoming connections, $x_{i}$ are inputs to the neuron, and f is called transfer function and should be selected according to the classification problem.

Neurons in a FNN are organized in layers: in the input layer, one neuron for each input variable is required; the number of neurons in the output layer is decided according to the number of classes to be recognized and the selected transfer function; between input and output layers a certain number of hidden layers can be inserted, whose dimensions are usually decided testing different configurations.

In this study we fixed a basic network structure with input layer and first hidden layer both including one neuron for each feature selected according to the first substring of the GA solution, and an output layer made up of one neuron returning the recognized activity. Then, the number of hidden layers was increased according to the second substring of each solution: three bits were used for adding from one to eight further hidden layers to the basic structure. Each new hidden layer included ½ of the previous layer neurons.

The sigmoid transfer function was used for all hidden layers and the linear transfer function was set for the output neuron. Since the output neuron retuned a real value for each classified element, the round operator was applied to the FNN output and used to assign the final class.

#### 3.4.3. Support Vector Machine

A SVM is a binary classifier (meaning that it is able to distinguish between two classes) that projects the input elements in a new multidimensional space, usually with higher dimensionality than the original one, in which the elements belonging to the two classes are linearly separable. The mapping from the original to the new space is accomplished by means of a function called kernel function, that could be linear or non-linear according to the problem complexity. In the new space, the separation between the classes is obtained with a hyperplane that maximizes its distance from the so-called support vectors. These are the elements of the two classes nearest to the hyperplane and their distance from the hyperplane is called margin.

For this kind of classifier, two different parameters must be set: the kernel function and the penalty term *C*, that regulates the tradeoff between large margins and small misclassification errors. Thus, we codified in the second substring both information, using two bits for choosing the kernel function and 4 bits for selecting the *C* value. For the kernel, we examined four different functions: linear, Gaussian, polynomial of order 2 and polynomial of order 3. For the penalty term, the value was set according to the following equation:

(4) $$C=\{\begin{array}{c} 0.5\;\;\;\;\;if\;C_{dec}=0\;\\ 1\;\;\;\;\;if\;C_{dec}=1\\ (C_{dec}-1)\times 10\;\;\;\;otherwise \end{array}$$

where $C_{dec}$ is the decimal value of the 4 bits codifying the *C* term. Using Equation (4) we were able to explore values between 0.5 and 140.

Since the SVM is a binary classifier and, in this study, we would like to identify seven different activities, we implemented a multiclass model for SVM. It combined 21 SVMs using the one vs one strategy in which, for each classifier, only two classes were evaluated, and the rest was ignored. In this way all possible combinations of class pairs were evaluated. The final classification is then obtained using the majority voting.

#### 3.4.4. Decision Tree

A DT is a tree-like classifier belonging to machine learning methods. In general, the tree is constructed top-down by recursively dividing the training set into partitions according to a given splitting rule in the form of “*if variable_i_ < threshold then partition1, else partition2*”. For each splitting rule, a new node is created in the tree. The best splitting rule is identified as that producing two partitions as pure as possible, where pure means that all the elements into a given partition belong to the same class. The construction of a branch stops when the obtained partition is pure or if no more variables can be used for partitioning: in case of pure partitions, the class of the elements is assigned to the leaf node, while in case of no pure partitions, the corresponding leaf node is labeled with the most represented class in the partition. During DT construction it could happen that not all available variables are used, thus a selection of the most discriminant features could be obtained as byproduct of this classifier. Once the tree has been constructed, a new element is classified iteratively applying the splitting rules and following the corresponding branch until a leaf node is reached: the class of the leaf is automatically associated to the new element.

Although several algorithms have been proposed for the tree construction and the identification of the best splitting rule for each node, the CART algorithm [55] and the Gini index [55] are commonly used for the these purposes, respectively, and applied in this study. Once these methods have been selected, no other parameters must be set for DT construction and running. For this reason, in our GA the optimization of the DT did not require bits associated to the second substring.

### 3.5. Post-Processing

Each couple feature subset-classifier parameters identified by GAs was used for classifying all dynamic windows in the validation set. Furthermore, a post-processing algorithm based on majority voting was implemented on the outputs of each classifier, to reduce isolated classification errors: considering 5 subsequent windows, the most frequently recognized activity was assigned to the entire group of 5 overlapping windows.

### 3.6. Performance Evaluation

The performances of the eight couples feature subset-classifier parameters were evaluated in terms of accuracy reached for each dynamic activity in every subject involved in the study, after post-processing. The results obtained for *FeatSet_A* and *FeatSet_B* across the 61 subjects were also compared by means of a Student t-test (paired, 2-tail, significance level: α = 0.05), for each activity separately. Moreover, the *F1-score* [56] was calculated for each classifier as:

(5) $$F1-score=\frac{2\times precision\times recal}{precision+recall}$$

where *recall* measures the ratio between the number of true positive elements and the total number of positive elements and *precision* measures the ratio between the number of true positive elements and the total number of elements classified as positive.

## 4. Results

A total of 59780 windows were included in the validation set (61 subjects × 140 windows × 7 activities).

The separation of static activity windows from dynamic activity windows based on rules was able to correctly detect 100% of resting windows and 100% of upright standing windows. Thus, for GA implementation and performance evaluation, 42,700 dynamic windows were used as validation set and 4270 windows were randomly included in the training set.

Table 1 summarizes the GA results for each classifier and for the two feature sets. The following information, related to the best solution found by GAs, are reported: number of features selected by the first substring, classifier parameters codified in the second substring, accuracy obtained on the training set used for the classifier construction, and accuracy reached on the validation set comprising all dynamic windows.

As it emerges from the table, the GA allowed a substantial reduction of the number of features that was almost halved for both feature sets. Except for DT, a higher number of variables were selected from *FeatSet_B* with respect to *FeatSet_A*, even if this was not associated to substantial differences in the classifiers parameters and this did not produce better performances, neither on training nor on validation set.

**Figure 3:**
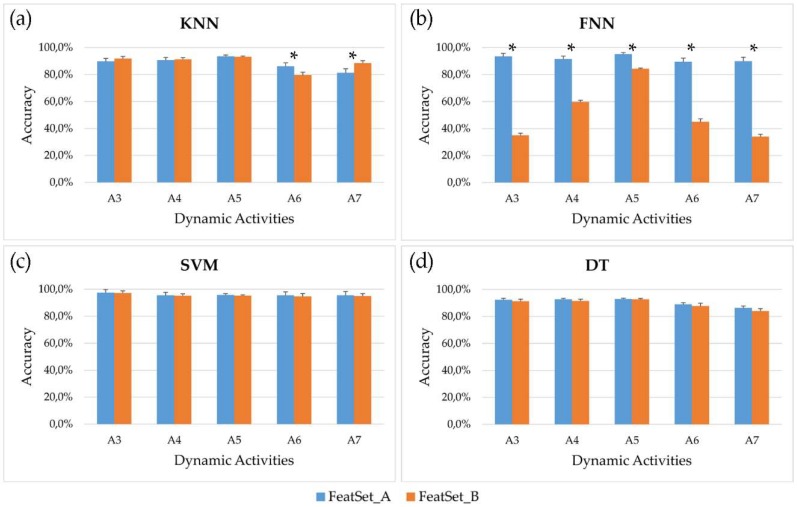
Mean accuracy (bar) and standard error (whisker) across the 61 subjects involved in the study for each dynamic activity (level walking (A_3_), ascending and descending stairs (A_4_ and A_5_), uphill and downhill walking (A_6_ and A_7_)), after post-processing. Four classifiers are analyzed: (**a**) K-Nearest Neighbors; (**b**) Feedforward Neural Networks; (**c**) Support Vector Machine; (**d**) Decision Tree. Asterisks (*) mark significant differences between accuracies reached by *FeatSet_A* and *FeatSet_B* (*p*-value < 0.05).

Figure 3 shows, for each optimized couple feature subset-classifier parameters, the mean accuracy and the standard error across the 61 subjects involved in the study after post-processing.

**Figure 4:**
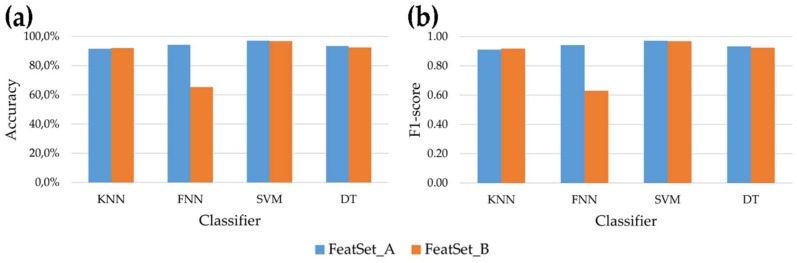
Mean accuracy (panel (**a**)) and F1-score (panel (**b**)) of the four classifiers across the seven activities (both static and dynamic activities), after post-processing, for the two sets of features.

Analyzing the behavior of the four classifiers, it emerges that the SVM reached the best performances, allowing to correctly recognize more than 95% of windows for all dynamic activities and for both feature subsets. Comparing the two feature subsets, no significant differences were observed for every dynamic activity using SVM and DT, while FNN fed with *FeatSet_B* was not able to reach acceptable results. This behavior is also evident in Figure 4, where the mean accuracy and F1-score across all seven activities (both static and dynamic) examined in this study is showed for each classifier. Overall, the highest accuracy achieved by the SVM is 97.1% and 96.7% for *FeatSet_A* and *FeatSet_B* respectively, while the worst mean accuracy was 65.5% obtained using FNN fed with *FeatSet_B*. The same behavior can be observed for the F1-score (Figure 4 panel b): the SVM had a score equal to 0.971 and 0.967 for the two sets, meaning that very high values of recall and precision were reached in both cases.

## 5. Discussion

In this work we compared two sets of features for HAR applications, one comprising features widely used in literature for similar purposes [24] and the second set including variables connected to the expected biomechanical meaning of a given activity on the sensor signals.

With respect to the *FeatSet_A*, our results are in accordance with or better than those obtained by other similar studies. In a recent study by Yurtman et al. [41], the authors compared seven machine learning classifiers using only time- and frequency-domain features. Their results showed that, considering both static and dynamic activities, the best classifier was the SVM, that allowed them to recognize the 86.4% of activities. Similarly, in our study the best results were achieved by the SVM, although our mean accuracy across the seven examined activities was 97.1%. Moreover, our methodology allowed to correctly recognize all windows related to static activities (accuracy = 100%), whereas in ref. [41] better performances were obtained for non-stationary activities with respect to stationary ones (accuracy of 90.7% and 70.6% respectively). Attal et al. [8] analyzed the total accuracy across static and dynamic activities of different classifiers and they found that the best method for HAR was the KNN, that reached 99.3% of correct classification. In our case, the mean accuracy of KNN across the seven examined activities was 91.6% but this was our worst result for *FeatSet_A* (see Figure 4 panel a).

Regarding the second set of variables, sometimes defined as heuristic features [14], it was rarely used for activity classification thus a comparison with previous studies is difficult. Reference [1] used some heuristic variables, such as zero crossing rate and peak-to-peak amplitude, in combination with other time-domain and frequency-domain features. The gyroscope signal integration was used in the study by Najafi et al. [57] for identifying postural transactions and further processed using the discrete wavelet transform. However, to the best of our knowledge, no studies proposed an entire set of features context-based. In our study, these variables were defined with the support of an expert in movement analysis that analyzed in detail the acquired signals during different types of activity and the expected biomechanical effect of a given activity on the sensor signals. From our results it is evident that this kind of variables, associated with the proper classifier, can effectively be used for HAR purposes with very good results (mean accuracy above 96% when used in combination with SVM). From the implementation point of view, the computational complexity of this set of features is lower than the one required by frequency and time-frequency domain features [15], since no transformation of the signals is needed and features are extracted only in time-domain. Moreover, having a direct physical meaning, heuristic features can be useful for supporting the interpretation of results in more complex situations, for example in the monitoring of pathological subjects. In fact, in presence of pathological conditions, the acquired MIMU signals could be altered and consequently some of the extracted features could differ from a “standard” condition. In this case, using *FeatSet_B* and analyzing the physical meaning of these “altered” variables, it could be possible to understand which biomechanical aspect is mostly compromised by a given pathology.

Finally, our study is the first in the HAR field in which the simultaneous optimization of feature subset and classifier parameters was performed. This allows to effectively obtain the optimal combination between input variables and classifier. Moreover, the dimensionality reduction obtained with GA allows for removing redundant and irrelevant features for the initial set of variables, preserving the feature meaning and supporting the results interpretation. On the contrary, other methods widely used in HAR literature, such as PCA [41] or linear discriminant analysis (LDA) [58], produce a transformation of the original variables that could complicates the understanding of the obtained results.

The main advantage of the proposed methodology is that the feature subsets were compared to the best of their performances. In fact, since a wrapper FS method was implemented with GAs, the optimal reduced subset was identified in both situations. Moreover, the simultaneous optimization of the classifiers allowed to find the proper set of parameters suitable for that specific input features.

One limitation of this study lies in the fact that only healthy subjects were involved in our experiment. However, our aim was to compare the two sets of features, thus the most basic and common situation was used, with no introduction of gait variability due to pathological conditions. Nevertheless, we are planning to enlarge our protocol to other neurological pathologies such as Parkinson disease. Moreover, we are implementing the HAR directly on a wearable device composed of three MIMU sensors (accelerometer, gyroscope and magnetometer) and a 32-bit microprocessor equipped with floating point processing unit. The optimized version of the SVM associated with the selected *FeatSet_B* variables was chosen to be implemented on this new device version.

## 6. Conclusions

This study focused on the emerging field of HAR and aimed at comparing a set of variables commonly used in literature with a completely new one, comprising only time-domain variables associated with the biomechanical meaning of acquired signals. Moreover, we used a methodology for simultaneous feature selection and classifier parameter optimization, based on GA and never used before in similar contexts. From our results it emerged that the two sets of features can both reach very high recognition accuracy, above 96%, if associated with the SVM classifier. However, the newly-proposed set of variables can be easier to be interpreted and their biomechanical meaning could be employed to better understand alterations of the biomechanical behavior in more complex situations, such as when applied to pathological subjects.

## Figures and Tables

**Table 1:** GA results for each classifier and for the two feature sets.

Classifier	# of Selected Features		Classifier Parameters		Accuracy on Training Set		Accuracy on Validation Set	
	*FeatSet_A*	*FeatSet_B*	*FeatSet_A*	*FeatSet_B*	*FeatSet_A*	*FeatSet_B*	*FeatSet_A*	*FeatSet_B*
**KNN**	106	132	*K* = 55	*K* = 55	87.7%	86.6%	87.7%	86.1%
**FNN**	114	138	*#hidden layers =* 6 *#hidden neurons =* [114, 57, 29, 15, 8, 4]	*#hidden layers* = 6 *#hidden neurons =* [138, 69, 35, 18, 9, 5]	91.7%	49.7%	89.7%	48.5%
**SVM**	118	133	*kernel =* gaussian *scale* = 20	*kernel =* gaussian *scale* = 10	100.0%	99.9%	98.5%	96.4%
**DT**	151	103	None	None	97.7%	97.1%	85.9%	82.7%
